# *Modular Adaptive Processing Infrastructure* (*MAPI*): a blueprint for interconnecting generic workflows with modern interfaces

**DOI:** 10.1107/S1600577525009269

**Published:** 2026-01-01

**Authors:** Aljoša Hafner, George Kourousias, Marco De Simone, Roberto Pugliese

**Affiliations:** a Elettra Sincrotrone Trieste SS 14 km 163,5 in Area Science Park 34149Basovizza Trieste Italy; Paul Scherrer Institut, Switzerland

**Keywords:** data analysis, scientific computing, computed tomography, software architecture

## Abstract

In this paper, we introduce the *Modular Adaptive Processing Infrastructure* (*MAPI*), a comprehensive software suite and approach designed to streamline and enhance data analysis workflows in scientific research laboratories. *MAPI* selects and integrates multiple frameworks and toolkits into a web-based platform, offering a highly modular and adaptable solution for diverse data analysis requirements. By design, *MAPI* supports distributed processing across heterogeneous backends (edge workstations, on-premises servers, high-performance computing and public cloud), making it suitable for various beamlines and data-processing labs. This blueprint, or ‘recipe’, provides a flexible infrastructure that can be tailored to specific experimental needs. We showcase *MAPI*’s application through its successful implementation on the X-ray computed tomography (CT) beamline, resulting in a system for tomographic processing (*STP3*). The case study demonstrates *MAPI*’s effectiveness in meeting complex computational demands, highlighting its potential for widespread adoption in scientific research environments. Most of the results reported in the paper are from a production deployment on Elettra’s SYRMEP beamline using two on-premises GPU servers, but two additional ongoing deployments on different beamlines are discussed.

## Abstract 2

*MAPI* is a modular web-based framework designed to unite legacy analysis code with heterogeneous backends (edge, on-premises, high-performance computing, cloud), dramatically accelerating data analysis workflows and offering a scalable flexible blueprint for modernizing synchrotron data analysis.

## 1. From synchrotron and experiment controls to modular data analysis

From their early operation, synchrotron facilities have placed stringent requirements on the control systems managing their accelerator components. This necessity fostered the development of specialized supervisory control and data acquisition (SCADA) frameworks such as *EPICS* (*Experimental Physics and Industrial Control System*, https://epics-controls.org/) and *TANGO* (https://www.tango-controls.org/), with a focus on distributed control. By continuously evolving, these systems facilitated increasingly complex accelerator and beamline control. But as synchrotron technology advanced, so too did the complexity of beamlines, necessitating tailored approaches to data acquisition.

Consequently, new specialized data acquisition frameworks have emerged in recent years, notably *BlueSky* (Allan *et al.*, 2019 ▸; Basham *et al.*, 2015 ▸) and *DonkiOrchestra* (Borghes & Kourousias, 2016 ▸), alongside broader-purpose solutions like *LabVIEW* (https://www.ni.com/labview). These frameworks, built on accelerator control foundations, have enabled more sophisticated experiments and streamlined developmental workflows.

In parallel with advancements in data acquisition, scientific experiments have increasingly demanded sophisticated data analysis and computational capabilities. Modern research infrastructures now routinely perform analyses using powerful desktop hardware or dedicated high-performance computing (HPC) systems (Blaschke *et al.*, 2023 ▸; Zhang *et al.*, 2024 ▸). However, as experiments become more diverse and computationally intensive, traditional analytical tools often struggle to keep pace with evolving experimental needs. We anticipate the emergence of specialized data analysis frameworks analogous to those established for accelerator control and data acquisition. Specializing in crystallography workflows, *MXCuBE* (Gabadinho *et al.*, 2010 ▸; Oscarsson *et al.*, 2019 ▸), with its web-based interface component *MXCuBE-Web*, has proven to be very versatile and capable, being adopted by many facilities worldwide. However, *MXCuBE* is intended for macromolecular crystallography and is thus not readily extensible to other applications. The *Modular Adaptive Processing Infrastructure* (*MAPI*) introduced in this paper directly addresses this evolving need in a flexible and modular manner. Currently, data analysis workflows commonly employ standalone graphical user interface (GUI) applications for Windows and Linux, command-line programs, scripting environments (typically Python-based), interactive notebooks (such as *Jupyter*, *Mathematica* or *MATLAB*), and workflow-centric platforms like *Orange* (Demšar *et al.*, 2013 ▸) or *Tofu* (Faragó *et al.*, 2022 ▸). Additionally, specialized systems frequently exploit non-interactive job-submission queues on HPC clusters, executing precompiled command-line applications.

Modern high-throughput experiments further necessitate near-real-time data visualization for prompt feedback, interactive exploration and adaptive tuning of parameters [Basham *et al.*, 2015 ▸; Albers *et al.*, 2024 ▸; Pithan *et al.*, 2023 ▸; Bicer *et al.*, 2017 ▸; Buurlage *et al.*, 2019 ▸; Konstantinova *et al.*, 2022 ▸; *HTTomo* (*High Throughput Tomography* pipeline, https://diamondlightsource.github.io/httomo/index.html)]. Frequently, these tasks are most effectively managed through web-based interfaces, a choice driven by accessibility, ease of collaboration and cross-platform compatibility, as detailed in subsequent sections.

**Figure 1:**
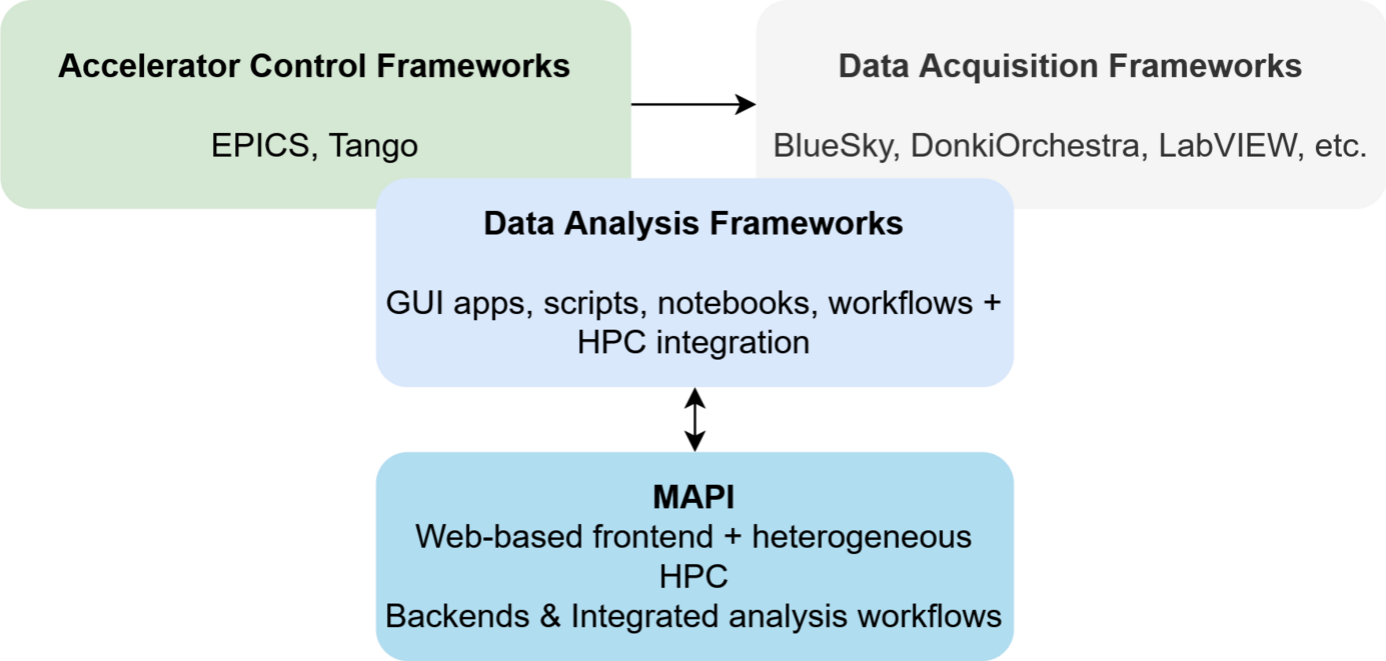
Specialized for synchrotrons, distributed control systems enabled advances in both accelerator control and beamline experiment data acquisition. *MAPI* aims to provide a broad framework for assisting in analysis of the acquired data and integrates various established approaches to computation.

The *MAPI* approach integrates established analytical methods within a unified architecture that couples a web-based frontend with heterogeneous computational backends. In this article we validate *MAPI* on two on-premises high-end GPU rack-mounted servers configured as a Beowulf HPC Linux cluster. Cluster load and job distribution are managed by *MAPI* with a custom Python implementation using *Celery* (https://docs.celeryq.dev/) and *Redis* (https://redis.io). Compatibility with edge devices, clusters and public cloud is provided by the architecture but not exercised in the reported results. As shown schematically in Fig. 1 ▸, *MAPI* not only bridges existing gaps but also significantly accelerates the prototyping and deployment of flexible customizable data analysis workflows, thus reducing development and integration overhead. Its innovation lies in its inherent flexibility and adaptability. It is designed from the outset as a generic framework capable of accommodating diverse experimental techniques. Subsequent sections of this paper provide a detailed overview of the *MAPI* architecture, highlight its key characteristics, discuss its software technologies and present an example use case demonstrating its application in a contemporary X-ray computed tomography (CT) data analysis system.

## 2. *MAPI* architecture at a glance

### 2.1. Architecture and software used

**Figure 2:**
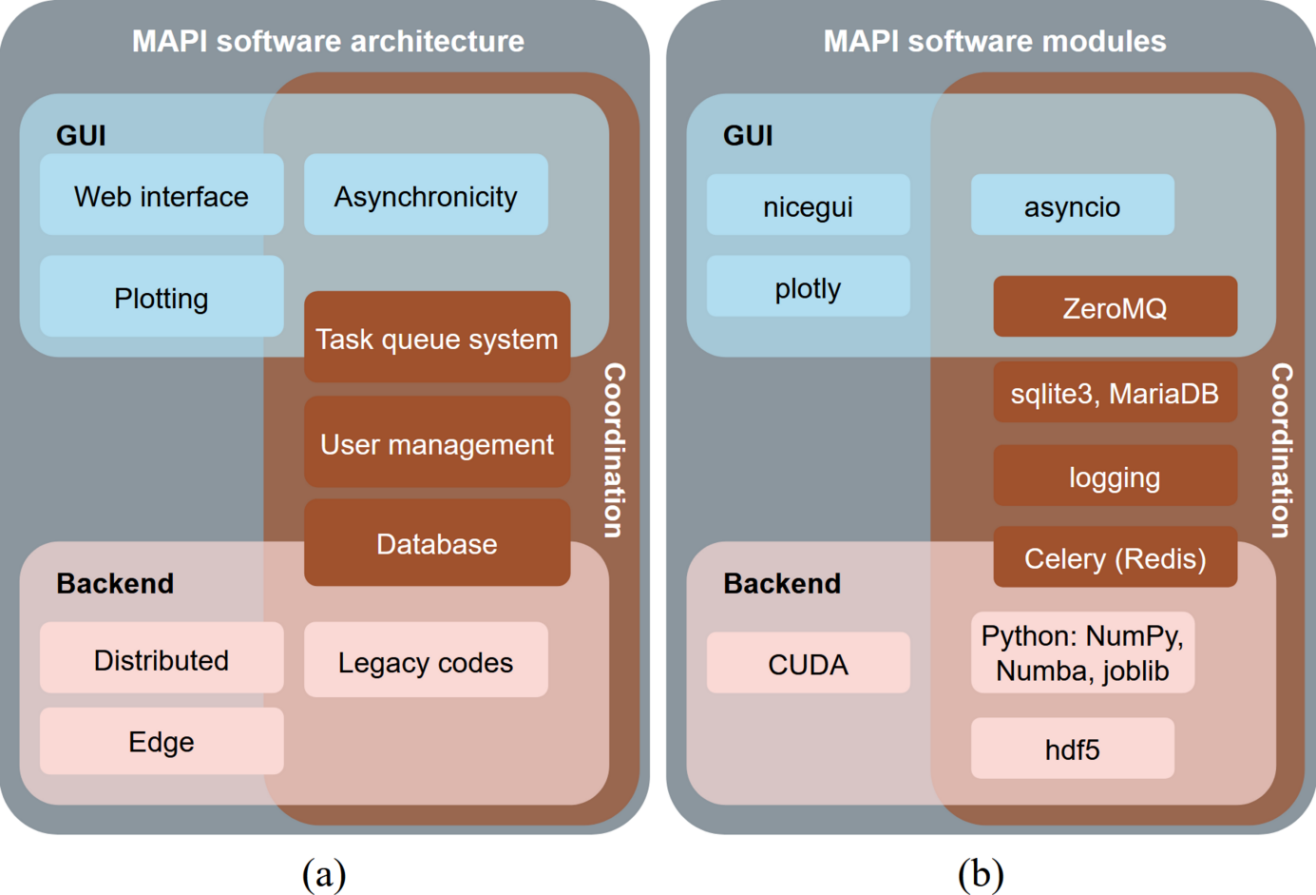
(*a*) Software architecture components consist of a frontend (GUI) part and the coordination layer (database + message broker). Backend functionality is then implemented as wrappers to the specific programs needed. Labels such as ‘Asynchronicity’, ‘Distributed computing’ and ‘Edge computing’ denote system properties, not software components. ‘Plotting’ refers to frontend visualization libraries such as *Plotly*. The coordination layer denotes the state or coordination (job metadata and parameters) persisting in the database and exchanged via the task queue. (*b*) Specific technologies used in the example application, covering the frontend, coordination layer and backend.

The software architecture is described briefly in Fig. 2 ▸(*a*). We use property labels (*e.g.* asynchronous execution, distributed and edge computing) to indicate behavior and placement, while the components are the frontend, the coordination layer and the backend wrappers. The primary focus and motivation throughout the development are aimed at the modernization of traditional data analysis workflows which mostly use legacy but effective code on individual workstations. Development has been centered around four main principles:

(i) a unified and easy-to-maintain web-based GUI with remote access from any platform;

(ii) distributed computing and hybrid setup (*e.g.* cloud, HPC, workstation or a mix);

(iii) integration into the existing facility user management system and data repository;

(iv) seamless usage of legacy backend analysis programs.

These principles have been distilled from our thorough analysis of modern use cases, and they address the main drawbacks of currently used programs.

For a GUI with platform-agnostic remote access, a web-based interface which allows for asynchronous execution has been selected. The frontend technology stack has to be selected in such a way as to be compatible with the existing facility user management system, allowing for secure user management and access to remote data repositories. The user management system integration and unified authentication are not just conveniences but are crucial for seamless usage of the whole (storage and computing) infrastructure. At Elettra, authentication is provided by the facility’s *Virtual Unified Office* (*VUO*), with remote access over the facility VPN or through dedicated HTTP tunnels initialized through *VUO*. *MAPI* uses *VUO* for user authentication and authorization (AAI) via a custom Python library (*pyvuo*) based on *FastAPI*. Together, the interface and tight user management integration make remote access possible ‘by design’ or via a custom HTTP reverse proxy tunnel created on the fly and used by Elettra’s user base, or via VPN access for internal staff. Alternative approaches to authentication can be from standard providers (*e.g.OracleDB*) or equivalent existing on-site systems, since *MAPI* does not have a hardcoded/integrated system but is designed to stay reliably behind one.

In contrast to traditional HPC systems with a scheduler [*e.g.SLURM* (https://slurm.schedmd.com)], we should allow users to launch jobs directly from the interactive frontend described above and for the jobs to be launched automatically. In this case, the frontend should be a full-featured analysis system which allows for efficient assessment of the data. Once a suitable set of parameters is found, the job is saved into a database where it can be consumed by a task queue system. The asynchronicity of the interface and decoupling of the frontend and backend allow for straightforward execution of legacy code in the backend.

As the architecture follows a modern web-application pattern, application-level coordination (such as job parameters and state between the front- and backends) persists in the database, while individual services internally communicate via standard HTTP APIs. The database thus acts as the point of truth for the job state, not as a network transport. The job execution is done by employing simple high-level wrappers (quasi-API) that follow the syntax of a selected task queue system. The jobs themselves should internally be able to communicate directly with the database, reading the selected parameters. The database is the crucial element that enables both the distributed computing capability and lag-free multiple simultaneous users.

Following the blueprint described above, concrete technologies used in our case are discussed and listed and shown schematically in Fig. 2 ▸(*b*). Since the popular JavaScript-based web technology stack is not readily used in scientific environments, we have chosen the more familiar Python-based web interface library *NiceGUI* (Schindler & Trappe, 2025 ▸). This allows for interactive data analysis work in a rich *Material UI* interface with *Plotly* (https://plot.ly) taking care of the plotting functionality. Furthermore, since *NiceGUI* uses *FastAPI*, there are no constraints on integrating the user management with the existing facility-wide database (*Oracle DB*) for which we already have a *pyvuo* Python API. In our deployment, authentication and authorization are provided by the *VUO* service. *MAPI* itself stores no credentials, and the thin integration layer can be swapped to other identity and access management (IAM) systems or databases without changing the application logic.

To address distributed computing and efficient usage of on-premises hardware, we have implemented *Celery* (*Distributed Task Queue*, https://docs.celeryq.dev/en/stable/) as our task queue system with a *Redis* message broker, but even simpler solutions are possible, *e.g.* a simple Python subprocess .popen call, giving up automatic horizontal scaling. Upon adding a new entry into the database, the job ID is sent to the *Celery* internal queue and thus delegated to an available worker. Usage of *Celery* makes writing the high-level worker wrappers simple and straightforward, as it consists of using a few decorators and correct settings for the *Celery* application. This also permits edge workers/nodes, as any machine can join the *Celery* pool. Here ‘edge’ denotes worker placement close to the acquisition hardware. *Celery* provides interactive dispatch of jobs and does not replace a cluster scheduler. On shared HPC systems, the *Celery* worker would submit via the site scheduler (*e.g.SLURM*’s sbatch/squeue), while the database would record the job state. Resource fairness and QoS (quality of service) remain under the scheduler.

The coordination layer uses a *MariaDB* relational database, which, together with the *Redis*/*Celery* message broker, runs on a separate virtual machine (VM). Usage of nonrelational databases such as *MongoDB* or even *SQLite* is possible without losing any functionality. The system is robust and asynchronous with support for multiple independent ‘views’ of the database, *i.e.* through *NiceGUI* AG Grid module or *phpMyAdmin* installation.

### 2.2. Infrastructure deployment

**Figure 3:**
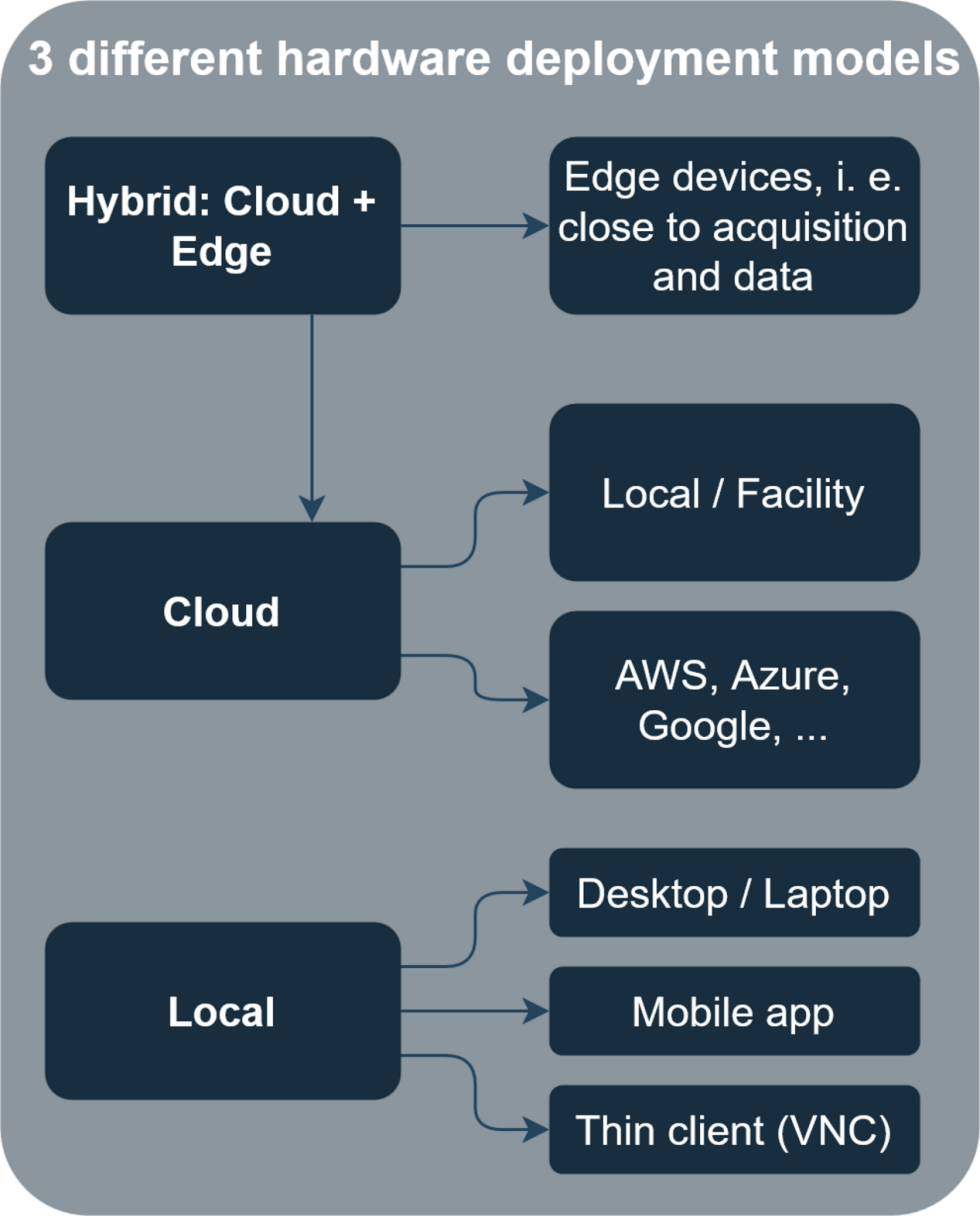
Three different infrastructure deployment models are foreseen. The first one (hybrid) is the suggested one for a large-scale facility which relies on both edge devices and existing computing infrastructure, and is described in this article.

*MAPI*’s infrastructural part is shown schematically in Fig. 3 ▸ and described and discussed below. Depending on the task, the tightly integrated existing facility user management allows users to access their own data (storage servers), perform analysis remotely and automatically obtain the correct permissions. Three different common infrastructure models are supported, although the present work evaluates only the first one:

(i) mixture of cloud, HPC and edge resources (external servers, internal servers and workstations);

(ii) cloud-only resources (only external servers);

(iii) local workstation mode.

The first case is the most common at scientific facilities, as the clients are usually workstations with various specifications and the desire is for calculations to be performed in-house, either on a workstation or on the HPC server. *MAPI* takes care of this by being deployed directly on said HPC server, which is able to serve clients through a web interface (managed by an *Nginx* or *Apache* web server installation). For the input/output (I/O) part, the storage servers must be accessible and mapped as local drives through either NFS, Ceph or similar solution. This way a user can analyze data from any device able to connect to the web interface. To avoid conflicts, we have implemented a simple in-time scheduler for GPUs which checks the available memory and directs the calculation to the GPU with the maximum amount of free memory, and imposed a limit on the number of concurrent workers in the *Celery* pool. Generally, on cluster deployments, comparable policies are typically enforced by the site scheduler. Our lightweight heuristic applies only to the two dedicated on-premises compute nodes described in Table 1 ▸.

In the case of heavy I/O, data acquisition should be done straight to the storage server and thus be decoupled from the analysis workflow. Such an infrastructure model also allows for edge workstations to be used as more than clients. Because all the parameters used in the calculation are passed from the interface to the database, one can select whether a local or remote database is used.

In the case of cloud-only resources, the model becomes both simpler and more complicated at the same time, as the database is located in the cloud without any edge devices. However, the actual I/O (transfer of data) between storage and calculation devices would be handled by *ZeroMQ* with a choice of two models, the subscriber–publisher model (allowing for several subscribers to a single publisher node) and a request–reply model (allowing for a more resilient connection).

Finally, *MAPI* also supports, by design, purely local deployment where the client, the database and any calculation servers all run on the same workstation, thus avoiding the need for any task queue system and using a simple *SQLite* database.

## 3. Use case application: Syrmep Tomo Project (*STP3 Web*)

X-ray CT at synchrotron facilities offers exceptional capabilities compared with laboratory systems. The SYRMEP (SYnchrotron Radiation for MEdical Physics) beamline at Elettra Synchrotron operates in the 9–40 keV energy range with monochromatic laminar beams covering up to 120 mm × 4 mm (Tromba *et al.*, 2010 ▸; Longo *et al.*, 2024 ▸). SYRMEP provides multi-scale imaging from sub-micrometre resolution (0.9 µm) to large field-of-view applications, supporting multidisciplinary research spanning biomedical applications, mater­ials science and cultural heritage studies. The beamline’s unique wide beam and long sample-to-detector distances (5 cm to 12 m) enable effective phase-contrast imaging, dramatically improving soft-tissue contrast and dose efficiency.

CT involves irradiating samples with X-rays while recording 2D projections as the sample rotates. On SYRMEP, typical experiments generate datasets with 2048 × 2048 pixel projections at 1800–3600 angular positions, creating 15–30 GB uncompressed HDF5 files. The reconstruction process utilizes the *ASTRA Toolbox* (Van Aarle *et al.*, 2016 ▸), a high-performance GPU-accelerated framework supporting various algorithms including FBP, SIRT, SART and CGLS with flexible geometries and efficient forward/backward projection operations.

### 3.1. *MAPI* implementation and architecture

**Figure 4:**
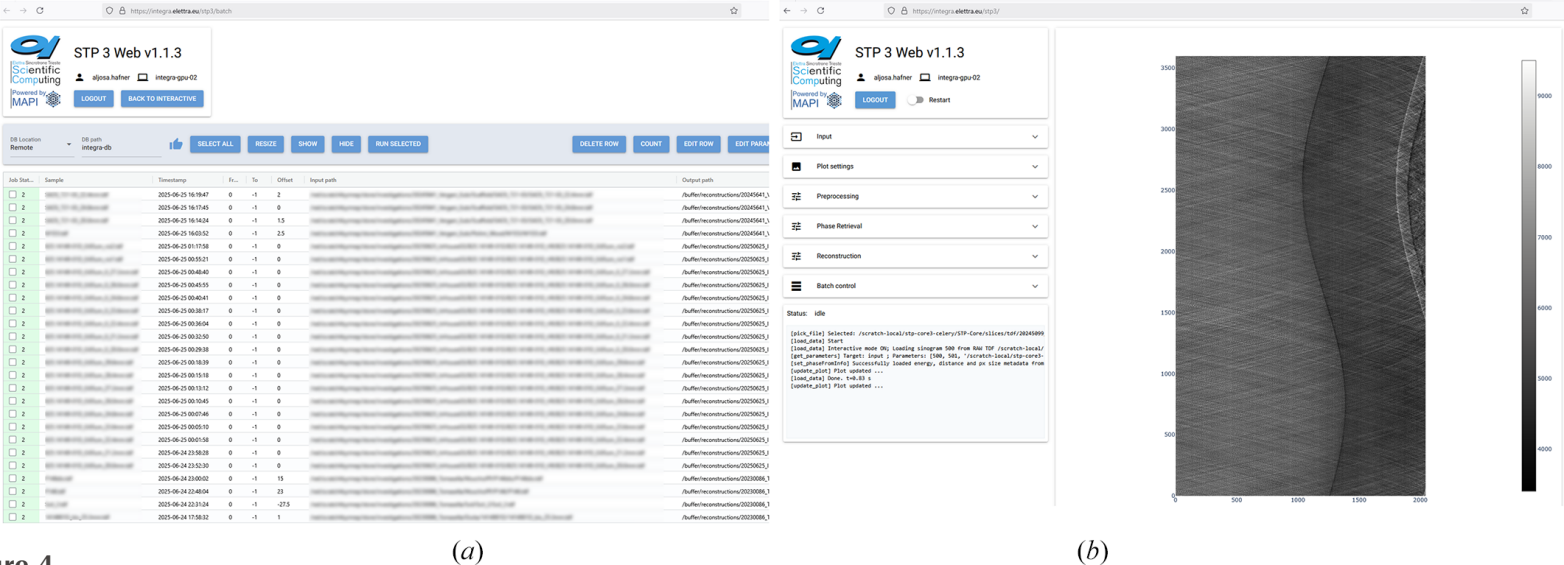
Two screenshots of the *STP3 Web* application in action. (*a*) A simple view of the remote database using the *NiceGUI* aggrid module. The user can inspect, edit and queue jobs from here. (*b*) The interactive mode, which allows for the selection of parameters and displays a preview of the final reconstruction. The parameters are then saved to the database from where they can be consumed by the broker and reconstruction performed on the full volume.

*STP3 Web* (Hafner *et al.*, 2025 ▸) represents the successful application of *MAPI* principles to address SYRMEP’s computational challenges. The system transforms traditional desktop-based workflows into a modern web-based platform while maintaining proven *ASTRA* reconstruction algorithms. Built using *NiceGUI* with *Material UI* components and *Plotly* visualization, the interface provides immediate access from any web-capable device, eliminating platform dependencies and enabling remote collaboration. Two screenshots are shown in Fig. 4 ▸.

**Figure 5:**
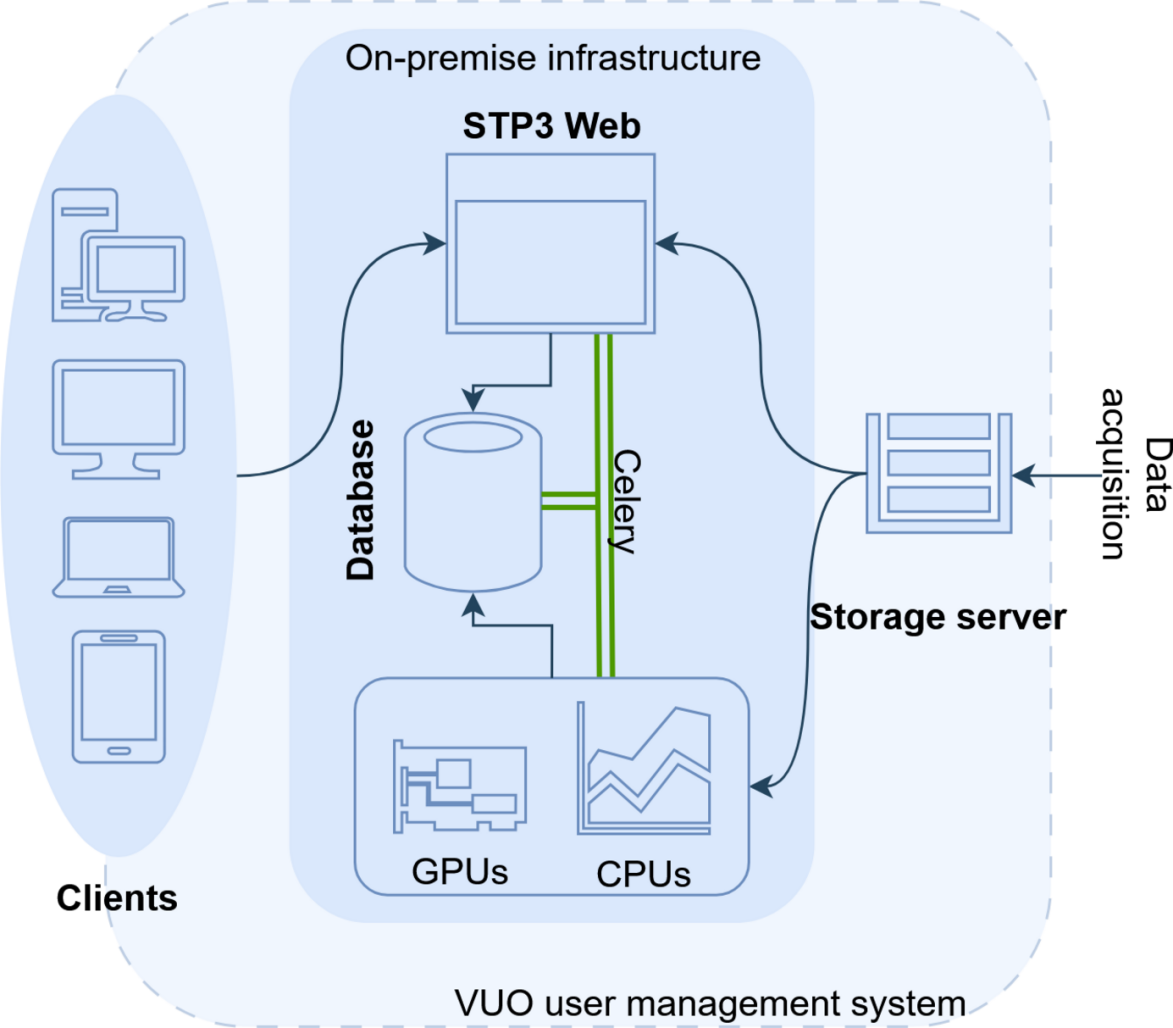
Current use case, *STP3 Web*. This is a CT data processing program which is built by partly reusing existing legacy desktop application code (backend and calculations) and the *MAPI* interface layer with the *Celery* intermediate queue and task management system. This allows each individual end user to use available computing resources directly from a website and ensures robust and secure operations. Legend: green lines (*Celery*) indicate job control/dispatch messages via the *Redis* broker and database pointers, and thin arrows indicate API calls and data paths (HTTP requests between services and file I/O between storage and workers).

As shown in Fig. 5 ▸, the architecture implements *MAPI*’s core principles through unified web interfaces, distributed computing via *Celery* distributed task queues, seamless facility integration including user management and data repositories (through Elettra’s *VUO*) and transparent utilization of existing reconstruction codes stemming from *STP* desktop application (Brun *et al.*, 2015 ▸; Brun *et al.*, 2017 ▸). In the schematic diagram, green *Celery* links represent control flow, while thin arrows show HTTP/API calls and bulk data movement. Job parameters are stored in a *MariaDB* database, enabling asynchronous execution where users interactively optimize parameters using preview sinograms before submitting full-volume reconstructions to available computational resources.

### 3.2. Performance and user experience

The transformation achieved through *MAPI* implementation has been dramatic. Interactive workflow speed improved by at least 600%, primarily through responsive interfaces, intelligent caching and optimized data handling (*e.g.* from 30 min to 5 min per dataset). Users can work with multiple datasets sequentially, saving parameters and immediately visualizing single-slice reconstruction previews. Batch reconstruction performance improved by 200% in sequential mode, with greater improvements possible through parallel execution. The improvements have been measured against the prior *STP* desktop workflow on the same datasets and hardware. For users, this translates to much quicker parameter-tuning loops with near-immediate single-slice previews, un­interrupted batch runs in the background and no manual steps (*e.g.* file conversions), addressing the main bottlenecks of the previous desktop workflow. We have already observed a significantly improved total data throughput of up to 4 TB per day per user.

These performance gains are achieved using identical backend reconstruction codes as mentioned by Brun *et al.* (2015 ▸, 2017 ▸), demonstrating *MAPI*’s effectiveness in workflow optimization. Further speed improvements stem from eliminating file conversion bottlenecks, optimized memory management, intelligent GPU scheduling and streamlined data paths. User feedback has been overwhelmingly positive, with particular emphasis on ease of use and responsiveness. The goal of enabling users to complete analysis by the end of their experimental session has been consistently achieved.

### 3.3. Infrastructure

**Figure 6:**
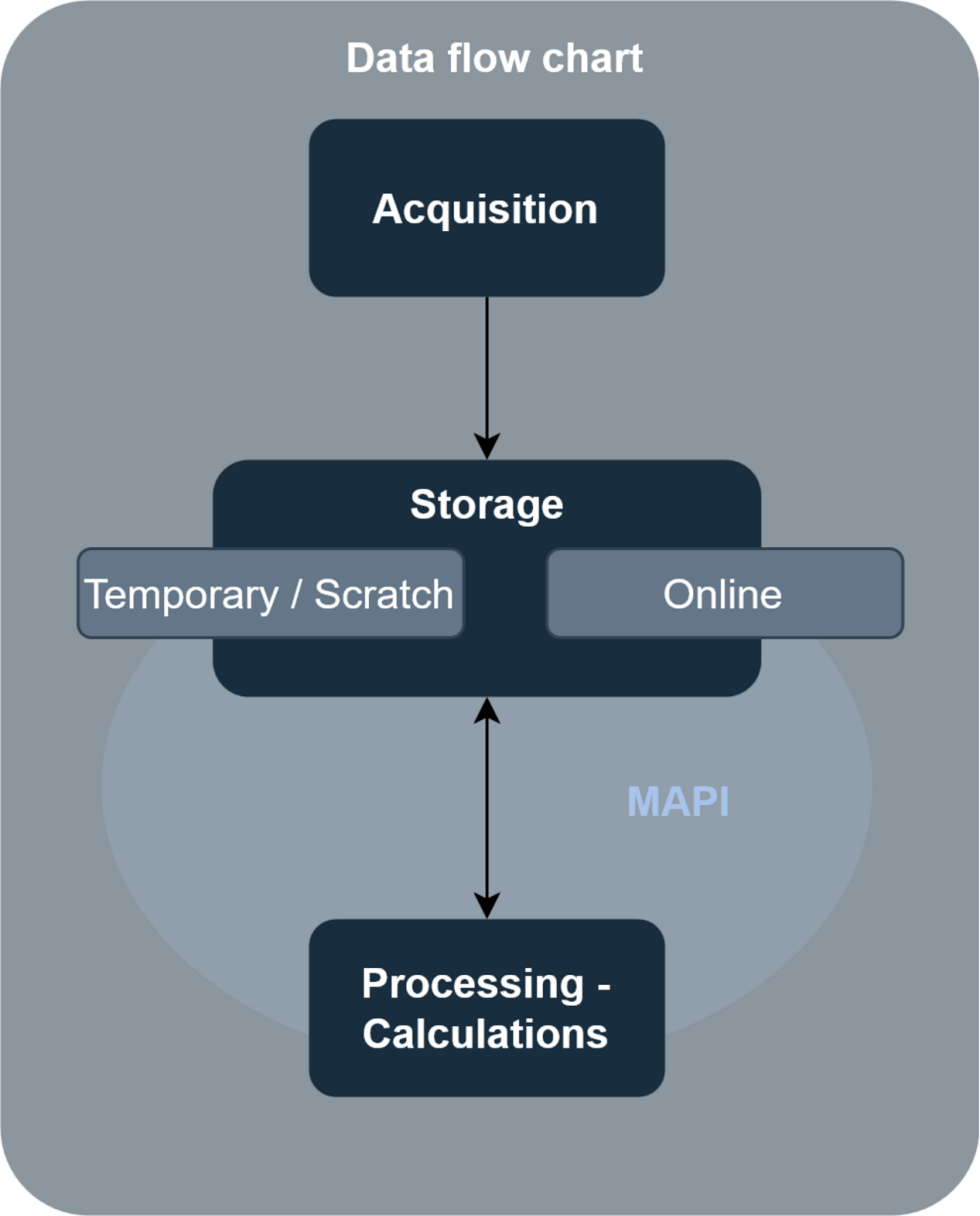
Simple data flow schematic. Data are generated by an edge device (acquisition) and transferred directly onto a temporary storage server. The data flows from and to the storage server are governed by the facility policy and individual user permissions. This allows for a seamless processing workflow without specifying the exact steps involved.

*STP3 Web* operates in full production at Elettra, serving multiple daily users including both internal researchers and external collaborators via VPN. The generalized data flow is presented in Fig. 6 ▸. The system handles approximately 3–4 TB of daily tomographic data processing, while maintaining a responsive performance for concurrent multi-user operations. Current deployment includes an INTEGRA compute node with 64 CPU cores, and six Nvidia Tesla T4 cards with 16 GB vRAM each and 1024 GB of memory, connected to the rest of the infrastructure by a 100 Gbit s^−1^ link. Recently, another node with an identical network connection of 100 Gbit s^−1^ was added, namely an INTEGRA 2 with 32 CPU cores, and one Nvidia L40S with 46 GB vRAM and 384 GB of memory. This has further enhanced the system capabilities and is summarized in Table 1 ▸. Everything is served by two NFS storage servers of 40 TB capacity, and two Ceph Rados object gateways. The Rados gateways make use of a dedicated pool of NVMe disks within our Ceph cluster, enabling scalable object storage. One NFS server is dedicated to data acquisition (HDFs) and another is used for the reconstructed images (individual TIFFs). The production environment demonstrates robust performance under real-world conditions, with redundancy and backup systems ensuring continuous operation during critical experimental periods. System health is continuously monitored 24 hours a day by *Zabbix* (https://www.zabbix.com/), providing real-time insights into performance and potential issues. All the code is deployed directly from our internal GitLab repository, ensuring version control and streamlined deployment. Raw experimental data, upon acquisition, is initially saved to an online storage solution utilizing a Rados device within a Ceph cluster. For enhanced data redundancy and disaster recovery, a duplicate copy of all raw data is also securely stored on our offline tape storage system. Finally, all the virtual machines have a daily scheduled backup.

### 3.4. Towards *STP4*

The development of *STP3 Web* required 18 person-months, encompassing both fundamental *MAPI* framework development and specific tomographic reconstruction implementation. This investment demonstrates the efficiency of the *MAPI* approach, as subsequent implementations are expected to require significantly less development time due to the established modular architecture and documented deployment procedures. Future developments in *STP4* focus on substantial enhancements driven by Elettra 2.0 (Gregoratti *et al.*, 2024 ▸; Karantzoulis, 2018 ▸) and SYRMEP LS (Life Science) beamline (Karantzoulis & Barletta, 2019 ▸) requirements, which will increase brilliance by at least 100× due to the significant reduction in horizontal emittance, and necessitate handling doubled or quadrupled data volumes. Planned improvements include advanced GPU memory management for larger datasets, distributed *ASTRA* implementations utilizing multiple computing nodes, machine learning-enhanced reconstruction algorithms, automated parameter optimization, and enhanced system stability through comprehensive monitoring and failover capabilities. The modular *MAPI* architecture ensures these enhancements can be implemented incrementally while maintaining continuous operation during facility upgrades.

## 4. Strategic developments and future prospects

The transition from the current synchrotron Elettra to Elettra 2.0 (Karantzoulis, 2018 ▸), which occurred during the submission of this article (final Elettra shutdown on 02/07/2025), marks a pivotal moment for synchrotron-based data analysis infrastructure. While Elettra 2.0 promises significant improvements in beam characteristics and beamline end­station capabilities, these advances, combined with progress in detector technology, are expected to generate data flows exceeding ten times current volumes, far beyond the improvements anticipated from hardware advances alone following Moore’s law and thus risking a data deluge. This dramatic increase in data throughput necessitates robust scalable solutions that can evolve with the facility’s enhanced capabilities. *MAPI*’s proven effectiveness through the *STP3 Web* implementation positions it as the foundation for addressing these future computational demands, with planned upgrades for successive *STP* versions supporting the enhanced future SYRMEP beamline (at Elettra 2.0) representing only a fraction of the broader deployment strategy.

The most significant expansion of *MAPI*’s application scope lies in its planned implementation across diverse experimental techniques throughout Elettra 2.0’s beamline portfolio. Preliminary studies have already commenced for integrating *MAPI* with advanced techniques including ptychography, coherent diffraction imaging (CDI), scanning transmission X-ray microscopy (STXM), X-ray microscopy (XRM), multimodal atomic force microscopy (AFM) and small-angle X-ray scattering (SAXS). These preliminary investigations have yielded promising findings, demonstrating *MAPI*’s adaptability to the unique computational requirements and workflow patterns characteristic of each technique. The modular architecture that proved successful for tomographic reconstruction naturally extends to these diverse applications, enabling rapid deployment while preserving the proven backend integration capabilities that allow continued utilization of established domain-specific analysis codes.

Albeit *MAPI*’s most mature adoption is *STP3 Web*, two more applications for two different beamlines and techniques are in active development. Focused on the SAXS beamline of Elettra (operated by TU Graz, Austria), an analysis system called *SashelWeb*, with a backend based on the *Sashel* (Burian & Amenitsch, 2018 ▸) binary package, has been devised and is in its specification stage. Similar to *STP3*, an interactive frontend gives remote and on-site users seamless access to computing resources while using legacy computational code. An expansion is planned into a fully fledged SAXS analysis tool integrating *ATSAS* components as well (Manalastas-Cantos *et al.*, 2021 ▸). Since *Sashel* and *ATSAS* are not heavy on input data, the input being a simple .txt file, cloud [such as AWS (Amazon Web Services)] backend computation modality is also under investigation.

Furthermore, an event-triggered *MAPI* analysis pipeline (*MAGNETO* – *MAPI Gizmo for Event-Triggered Operations*) for the Magnedyn beamline at the FERMI light source (Malvestuto *et al.*, 2022 ▸) is under active development. The analysis pipeline is more streamlined, as a series of pre­processing operations need to be performed on the raw data, and thus it does not require a rich interface. We have used integration with the existing *TANGO* control system and designed a minimal but powerful *RestAPI* which feeds the necessary incoming data parameters (path to raw detector data, preprocessing parameters, time, path to reduced data *etc.*) into a *MariaDB* database and triggers the Python-based backend, which is designed around a *Celery* task queue on the internal Elettra cluster. A frontend similar to the table view shown in Fig. 4 ▸(*a*) is implemented, showing the job status, basic manipulation and overview. The vision of the development team, facilitated by *MAPI*’s modular architecture, is its adoption by other beamlines and laboratories beyond Elettra.

Beyond synchrotron radiation technique-specific deployments, ongoing developments are focused on enhancing *MAPI*’s core capabilities through integration with emerging technologies. The most notable advancement involves integration with local/on-site large language model (LLM) services accessible through https://eai.elettra.eu, enabling intelligent command and control interfaces, automated log analysis, contextual help systems and intelligent processing parameter optimization. This integration represents a significant step toward autonomous data analysis workflows that can adapt to experimental conditions and user expertise levels. Additionally, *MAPI*’s proven framework has been incorporated into recent European Union project proposals focused on advanced materials design, highlighting its potential for cross-facility collaboration and standardization of analysis workflows across the broader European research infrastructure landscape.

The broader impact of *MAPI* extends beyond Elettra’s immediate requirements, with the framework designed for adoption by other institutes and commercial partners as needed. The blueprint approach, emphasizing adaptable architectures rather than rigid technological specifications, facilitates implementation across diverse computing environments and experimental requirements. As large-scale scientific facilities worldwide face similar challenges in managing increasing data volumes while maintaining user accessibility and computational efficiency, *MAPI*’s demonstrated success at Elettra provides a validated pathway for modernizing scientific data analysis infrastructure. This scalability, combined with the framework’s emphasis on integrating existing analysis code with contemporary web technologies, positions *MAPI* as a foundation for the next generation of collaborative distributed scientific computing environments.

## 5. Conclusions

The *Modular Adaptive Processing Infrastructure* (*MAPI*) provides a practical approach for scientific facilities to modernize data analysis workflows while preserving existing computational investments. As demonstrated through the successful deployment of *STP3 Web* on Elettra’s SYRMEP beamline, *MAPI* addresses an important need in scientific computing: frameworks that can integrate legacy analysis code with contemporary web-based interfaces and distributed computing capabilities.

The value of *MAPI* extends beyond its technical achievements. While the 600% improvement in interactive workflow speed and 200% enhancement in batch processing performance demonstrate measurable benefits, the framework’s modular architecture offers broader advantages for scientific facilities seeking to adapt their data analysis infrastructure. Like other specialized frameworks that have proved successful in accelerator control and data acquisition, *MAPI* provides a foundation for distributed collaborative data analysis that addresses evolving facility requirements.

The development of *MAPI* proves timely as synchrotron facilities prepare for substantial increases in data throughput. Elettra 2.0’s anticipated tenfold increase in data volumes exemplifies challenges emerging throughout the scientific community, where traditional analysis approaches may become limiting factors. *MAPI*’s modular architecture, validated through daily production use processing 3 TB of tomographic data, demonstrates that scalable solutions can be implemented while maintaining existing computational workflows.

MAPI’s blueprint approach emphasizes adaptability through its modular design rather than prescriptive technological choices. This architecture enables deployment across diverse experimental techniques, from ptychography to small-angle X-ray scattering, while maintaining core capabilities: facility integration, distributed computing and transparent utilization of domain-specific analysis programs. The ongoing integration with emerging technologies, including local large language models, demonstrates the framework’s capacity to evolve alongside advancing scientific requirements.

As research infrastructures face similar computational challenges, *MAPI*’s successful implementation at Elettra offers a practical pathway for modernizing scientific data analysis workflows. The framework’s emphasis on collaboration, accessibility and computational efficiency makes it relevant beyond its initial deployment, providing a foundation for addressing the infrastructure needs of contemporary experimental facilities. Through its modular approach and proven performance, *MAPI* contributes to the ongoing evolution of scientific computing environments that support increasingly sophisticated experimental research.

## Figures and Tables

**Table 1:** Specifications summary of the two currently connected dedicated GPU compute nodes, configured as a Beowulf HPC Linux cluster used in *STP3 Web*

System name	Storage (TB)	Network (GbE)	CPUs	Memory (GB)	GPUs (TFLOPS FP32)
INTEGRA	2	100	2 × 32-core Intel Xeon Gold 6264R	1024	6 × Nvidia Tesla T4, 16 GB vRAM (∼49 TFLOPS total)
INTEGRA 2	14	100	1 × 32-core Intel Xeon Gold 6444Y	384	1 × Nvidia L40S, 46 GB vRAM (∼96 TFLOPS)

## Data Availability

The *MAPI* code is available under a CC-BY-NC 4.0 license in Elettra’s public GitLab repository,https://gitlab.elettra.eu/sciqc/stp-core3-celery.
